# Boosting the resolution of multidimensional NMR spectra by complete removal of proton spin multiplicities

**DOI:** 10.1038/s41598-021-01041-8

**Published:** 2021-11-03

**Authors:** Peyman Sakhaii, Bojan Bohorc, Uwe Schliedermann, Wolfgang Bermel

**Affiliations:** 1 NMR Laboratory of SANOFI, Global CMC Early Development, Synthetics Platform, Industrial Park Hoechst, Building G849, 65926 Frankfurt, Germany; 2 Bruker BioSpin GmbH, Silberstreifen 4, 76287 Rheinstetten, Germany

**Keywords:** Peptides, Structural biology, Chemical physics, Techniques and instrumentation

## Abstract

Over decades multidimensional NMR spectroscopy has become an indispensable tool for structure elucidation of natural products, peptides and medium sized to large proteins. Heteronuclear single quantum coherence (HSQC) spectroscopy is one of the work horses in that field often used to map structural connectivity between protons and carbons or other hetero nuclei. In overcrowded HSQC spectra, proton multiplet structures of cross peaks set a limit to the power of resolution and make a straightforward assignment difficult. In this work, we provide a solution to improve these penalties by completely removing the proton spin multiplet structure of HSQC cross peaks. Previously reported sideband artefacts are diminished leading to HSQC spectra with singlet responses for all types of proton multiplicities. For sideband suppression, the idea of restricted random delay (RRD) in chunk interrupted data acquisition is introduced and exemplified. The problem of irreducible residual doublet splitting of diastereotopic CH_2_ groups is simply solved by using a phase sensitive JRES approach in conjunction with echo processing and real time broadband homodecoupling (BBHD) HSQC, applied as a 3D experiment. Advantages and limitations of the method is presented and discussed.

## Introduction

Various types of multidimensional homo- and heteronuclear NMR experiments have found widespread use in many research and industrial laboratories. The vast diversity of NMR experiments has led to its successful application far beyond conventional structure elucidation purposes^1,2^. The practical implementation often starts with polarization transfer from proton and ends up with proton detection to have the maximum gain in sensitivity^3–7^. Due to proton detection, each signal is centered around its chemical shift δ and bears the spin multiplicity in the ω_2_ frequency dimension of the resulting 2D spectrum. The J multiplicities are the result of the continuous evolution of homonuclear scalar J coupling during signal detection. The quest for removing the spin multiplicities from NMR spectra to obtain only chemical shift information was raised in the early days of NMR by Hans Primas^8^.

Several strategies have been suggested over the years. The first strategy uses active decoupling during the evolution and/or acquisition period, i.e. the indirect or direct dimension^9–13^. Here most of the techniques are based on a proton spin subset, where a fraction of proton spins is decoupled from the rest of the spin reservoir. The second group of homodecoupling (HD) experiments is based on a full evolution of the J coupling in an extra dimension and a subsequent tilt to separate multiplicity and chemical shift in orthogonal dimensions^14–18^. The J-resolved (JRES) experiment is a prominent member of this family, which usually shows higher sensitivity since no subsets are involved. However, a real time HD version (decoupling in the acquisition dimension) of the JRES type experiment is not possible and an extra dimension must be used^19–22^. Other strategies are based on small flip-angles e.g. in a COSY (COrrelation SpectroscopY) experiment (anti-z COSY^23^) or a combination of small flip-angle and gradients e.g. in PSYCHE (Pure Shift Yielded by Chirp Excitation^24^). Since these strategies are not compatible with real time decoupling, they are not considered in the rest of the paper.

For the first group of active decoupling, a type of real time chunked decoupling can be used for HD in the direct dimension^25,26^. A block of dwell points is acquired, interrupted by the execution of pulses, delays and gradients (referred to as RF-block). While the time in which data points are sampled remains unchanged (apparent acquisition time), the total acquisition time is given by the apparent one and the length of all the RF-blocks. Since T_2_ relaxation also happens during the RF blocks, this difference will cause an additional line broadening depending on the T_2_ relaxation time. The resolution of the final homodecoupled (HD) proton spectrum is usually significantly increased due to the removal of proton spin multiplicities and thus the disentangled signal overlap. On the other hand, due to the proton spin subset constraints, the overall sensitivity can be reduced compared to a standard proton spectrum. Then resolution is gained on the cost of sensitivity. Proton spin subsets are created either by frequency (band selectivity)^27–29^, spatial (Zangger-Sterk)^11^ or ^13^C isotope selection^13,26,30,31^. Zangger and Sterk (ZS) first proposed an experiment, where a selective refocusing RF pulse in combination with a weak gradient pulse was applied to create the proton spin subset thus enabling a decoupling during the chemical shift detection in a direct or indirect dimension^11,25^. In ZS experiments, the resulting sensitivity is usually about 2% while the efficiency of the HD increases with the selectivity of the refocusing pulse. However, an unlimited decrease in bandwidth will ultimately increase the refocusing RF pulse length diminishing overall performance. Isotope filtration was proposed to overcome the bandwidth limitation by using an isotope filter. The BIRD (BIlinear Rotation Decoupling) isotope filter^30^ was applied in the indirect or direct dimension and delivered broadband HD NMR spectra even in cases of total spectral overlap. A homodecoupling could be achieved because the selective proton inversion is done topologically and is independent of bandwidth limitations. Due to natural abundance isotope distribution the sensitivity is similar to the ZS method. Yet since the BIRD element selects the same moiety as the HSQC as such no further loss in sensitivity is to be expected. Due to the structural topology of a CH_2_ group, where two protons are directly bound to the same ^13^C isotope, a residual doublet splitting survives the HD, because the BIRD filter is unable to refocus the J coupling of only one of the two protons. An example to combine BIRD isotope selection with a perfect echo filter applied in a pseudo dimension was published. The method delivered fully homodecoupled HSQC spectra at the expense of additional T_2_ relaxation^32^. The symmetrical shape of the perfect echo filter embedded into the BIRD filter requires the insertion of extra delays for a coherent ^2^J_HH_ refocusing. The resulting spectra showed a doubling of the natural linewidth. A comprehensive discussion about the concept of the BIRD type HD is given elsewhere^33^.

In the real time version of BIRD decoupling the physical acquisition is split into the BIRD building blocks and data chunks of distinct length. The periodic repetition of the construct refocuses the scalar J coupling and delivers the HD spectrum in the ω_2_ dimension. A close inspection of the so derived HSQC data shows residual sidebands around the singlet responses. Since T_2_ relaxation continues during the RF block, this will lead to steps at the interface of two chunks. This and imperfections in the refocusing of the homonuclear coupling will lead to the observed sidebands upon Fourier Transform^34^. Unfortunately, the sideband artefacts in ω_2_ are unaffected by phase cycling or ZZ-filters^35^ (applying gradients when all spins are along the z-axis) and deteriorate the quality of the HD spectra. Our ambition during the development of any new technique was to significantly reduce the presence of those sideband artefacts and remove the doublet splitting caused by the shortcoming of the BIRD filter for diastereotopic CH_2_ groups. The issue of sidebands also occurs in experiments where the decoupling is done in an additional dimension. Yet, we will focus on real time versions.

Earlier, the J resolved spectroscopy was proposed to deliver a HD proton spectrum without involving any proton spin subset^14^. In a two-dimensional experiment, chemical shift is exclusively evolving in the direct dimension while homonuclear scalar J coupling is active in both the indirect and direct dimension. After tilting the multiplets the final 2D δ, J-spectrum shows multiplicity information in ω_1_ and chemical shift in ω_2_. A projection along the ω_1_ dimension then results in a broadband HD 1D proton spectrum. One of the biggest advantages of the concept is that no spin subset is used and rather the entire proton spin reservoir is utilized. As a result, the JRES method delivers high sensitivity spectra compared to competitive techniques with the disadvantage that the spectra originally processed in magnitude mode showed unacceptable broad line shapes. Recently, an echo processing method was reported to bypass these difficulties and to obtain pure absorption J resolved spectra^19–22^. This yields phase sensitive J resolved spectra leading to high resolution broadband homodecoupled (BBHD) spectra in one or more dimensions^19,36^. A combination of the JRES technique with conventional HSQC executed as a 3D experiment is also conceivable to deliver phase sensitive fully homodecoupled spectra. However, like in conventional JRES experiments, a significant number of increments must be recorded to make sure that the lineshape after tilting is not dominated by the digital resolution in the t_1_ dimension. Even with non-uniform sampling (NUS) the total measurement time of such an experiment would set a practicability limit.

In this paper we propose a technique to significantly reduce the periodic artefacts in ω_2_ and completely remove the residual doublet splitting in HD spectra at high sensitivity. We combined the real time HD decoupling^11,25–29^ with a phase sensitive J resolved experiment exploiting the recently introduced echo processing to achieve this goal. Examples of fully HD HSQC spectra on Cyclosporine and a commercial mixture of 20 Terpene are used for illustration.

## Results and discussion

One of the main difficulties in obtaining real time BBHD HSQC spectra is the problem of sideband artefacts in the ω_2_ dimension, which are the result of alternating repeatedly between RF blocks and chunk data acquisition. In case of homonuclear ZS experiments, theoretical investigations were published to remove these artefacts by computational approaches and to obtain a reconstructed 1D sideband free pure shift spectrum^34^. Zangger et al.^37–41^ proposed to vary the length of the chunks in a random fashion to yield clean 1D pure shift spectra.

The original 2D HD HSQC uses a delay compensated BIRD^d^ element^26^. The application of a random variation of the chunk length as suggested for 1D ZS spectra produced an unacceptable high level of t_1_ noise. So, alternative approaches are needed. In addition to sideband suppression, we were also seeking to minimize the total number of pulse field gradient pulses (PFG) implemented within the BIRD element to reduce the risk of undesired echoes being recalled. In addition to the discussion about possible sideband artefacts, the original 2D BIRD HSQC experiment is able to homo decouple all proton multiplicities with the exception of diastereotopic CH_2_ groups. The remaining residual doublet splitting makes an unambiguous assignment rather difficult. To overcome this issue is another aim of this work.

**Figure 1:**
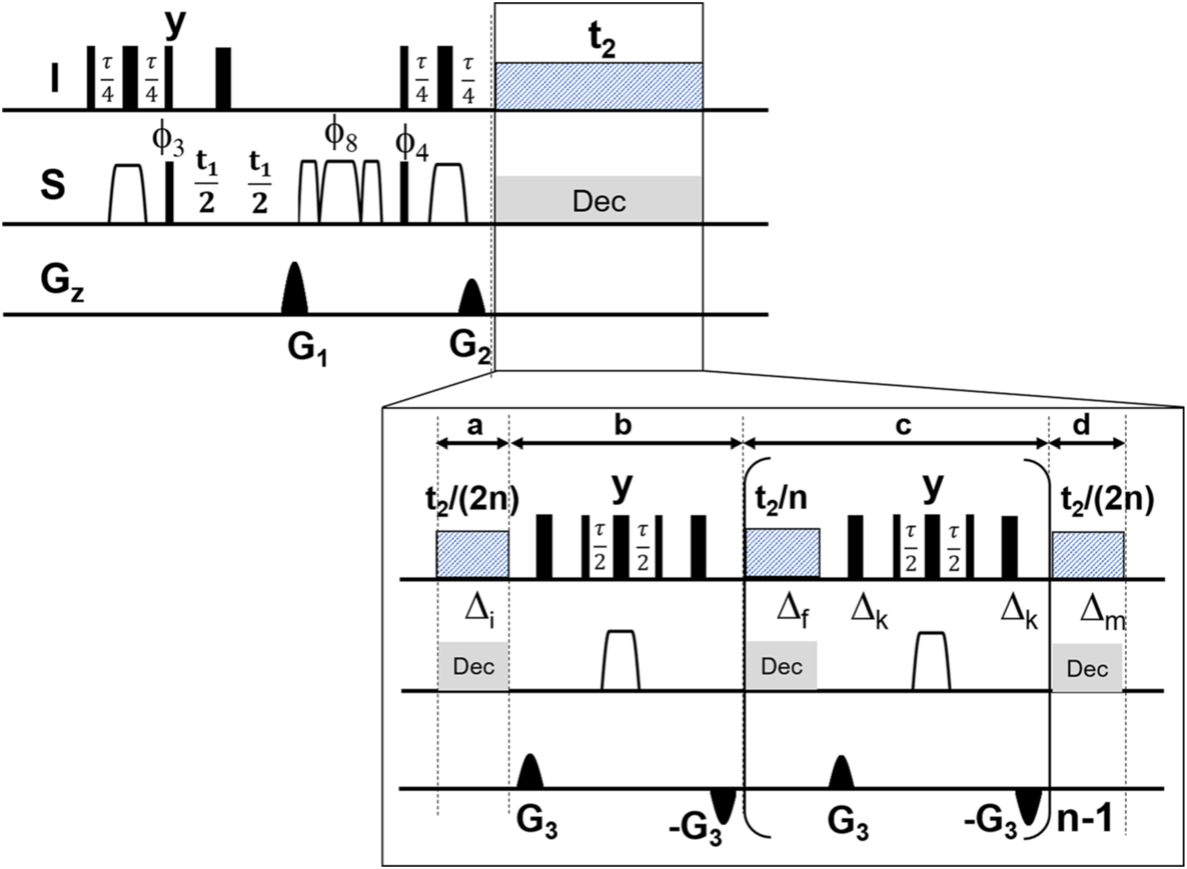
For obtaining 2D restricted random decoupled (RRD) BIRD HSQC spectra, the depicted pulse sequence is used. Proton spin multiplicities are removed by real time homodecoupling during the physical acquisition time using the indicated BIRD^r,X^ elements and chunk data blocks as shown in the insert. Details on experimental parameters are given below. Unless stated otherwise, pulses are applied along the x-axis. Narrow and wide filled rectangles represent non-selective ^1^H or ^13^C 90° and 180° pulses, respectively. The chirp pulses files are taken from the Bruker library: Crp60,0.5,20.1 is a smoothed chirp pulse for inversion with a pulse length of 500 µs and is shown as open trapezoid. Crp60comp.4^43,44^ is a composite chirp refocusing pulse with a pulse length of 2 ms and is shown as a triple of open trapezoids. The chirp pulses were defined with 1000 points and 4000 points, respectively, a sweep width of 60 kHz and 20% smoothing. The power of the chirp pulse was adjusted to a level equivalent to that of a 90° rectangular pulse of 25.5 µs (9.8 kHz), which corresponds to a Q factor of 5 (determined with Shape Tool). The pulsed field gradients indicated as filled sine envelopes are 1 ms and 0.3 ms in length with an amplitude according to a smoothed rectangle (10% smoothing on either side). The gradient files are taken from the Bruker library: SMSQ10.100 (1 ms) and SMSQ10.50 (0.3 ms). They are used for coherence selection (G_1_, G_2_) and artefact suppression (G_3_). The amplitudes of the gradient pulses have the following ratio: G_1_ = 80%, G_2_ = 20.1%, G_3_ = 35% (with 100% corresponding to 53.5 G/cm). The pulsed field gradients are applied along the z-axis followed by a gradient recovery delay of 200 us. The loop parameter n applied for the repeated BIRD decoupling was set to 12 leading to an initial chunk delay length of ∆ = 10.6 ms. This is a compromise between keeping the number of RF blocks small and at the same time not allowing too much J evolution to occur. The length of the chunk and decoupling blocks were varied by a limited number of randomly chosen delays between 0 and 10 ms, the exact list of delays employed is given in the Supplementary Material. The RRD HD HSQC spectrum was recorded using 8 scans (minimum of 4 scans) per increment and 2 s relaxation delay. The following delay parameter was used: τ = 1/(J_CH_) = 6.89 ms, the initial values for the t_1_ increment was 3 µs. Frequency discrimination in the ^13^C evolution dimension is achieved by an Echo/anti-Echo protocol. Broadband ^13^C decoupling was applied during the acquisition (t_2_) using a decoupler pulse length of 55 µs at a field strength (γB_1max_/2π of 4.5 kHz utilizing a garp4 decoupling super cycle given in the Bruker library^45^.

To achieve the goal of complete removal of proton spin multiplicities, we decided to work in 2 steps. First, we should diminish the sideband intensities usually present during the real time HD approach and second to remove the residual doublet splitting which is known to be unaffected by BIRD decoupling. For obtaining a significant reduction in sideband artefact intensities, we used the pulse sequence depicted in Fig. 1.

The pulse sequence consists of two parts, the first part describes a conventional HSQC sequence while the real time decoupling part is modified compared to literature^25,26^. A delay compensated BIRD^r,X^ pulse cascade is used as central element for the ^13^C isotope selection and HD. The delay compensation around the BIRD^r,X^ pulse was necessary to accommodate the timing for the PFG and switching commands for ^13^C decoupling. The symmetrical envelope of BIRD^r,X^ enables to reduce the number PFGs within a HD block.

Minimising the number of PFG pulses has an important impact avoiding accidental refocusing of coherences during the chunk data acquisition. This becomes evident when e.g. introducing an additional gradient into the zz period of the first INEPT (Insensitive Nuclei Enhanced by Polarization Transfer^42^) step which showed an increase in undesired signal rather than a reduction. Our first experimental results with the modified pulse sequence (Fig. 1) delivered sideband artefacts centered around the homodecoupled proton signals. The amplitudes of these artefacts are comparable to sequences known from the literature^25,26^. A modification was made to the length of all blocks present in the real time decoupling part (Fig. 1). The delay periods termed as a, b, c and d are chosen such that the length of those durations is randomly varied. Zangger et al. have successfully demonstrated the implementation of a truly random variation of the chunk length in the conventional ZS based 1D real time decoupling experiment. In our case, a direct transfer of the idea failed because it resulted in too much noise in the ω_1_ dimension. This is because in contrast to ZS 1D case, the varied delay length differs from increment to increment. This variation will lead to an extra and significant modulation of the FID in the ω_1_ dimension and hence result in a rather strong noise contribution in the ^13^C direction. We expanded this idea first to change the length of all delays a, b, c, and d using a limited number of randomly selected durations between 0 and 10 ms. This is the main difference to the previously reported concept. The limited number of truly randomly chosen durations is repeated for every increment so that no significant t_1_ noise is added to the 2D spectrum. The change of delay length is applied to all building blocks and we call this type of data acquisition Restricted Random Decoupling (RRD). The length of the first acquisition block (a, ∆_i_) is changed from scan to scan, while the durations within the loop (c, ∆_f_ and ∆_k_) are different for each loop. A detailed list of the delays in conjunction with the number of scans is given in the Supplementary Material. The RRD concept was also implemented into the original BIRD^d^ HD HSQC but due to a number of artefacts at specific frequencies in F_1_ it turned out to be inferior to the BIRD^r,X^ implementation. The combination of RRD with BIRD^r,X^ as depicted in Fig. 1 led to a reduction of sidebands with no significant t_1_ noise or other artefacts in F_1_. The larger number of spoil gradients in the original BIRD^d^ HD HSQC sequence is suspected to be responsible for these artefacts.

**Figure 2:**
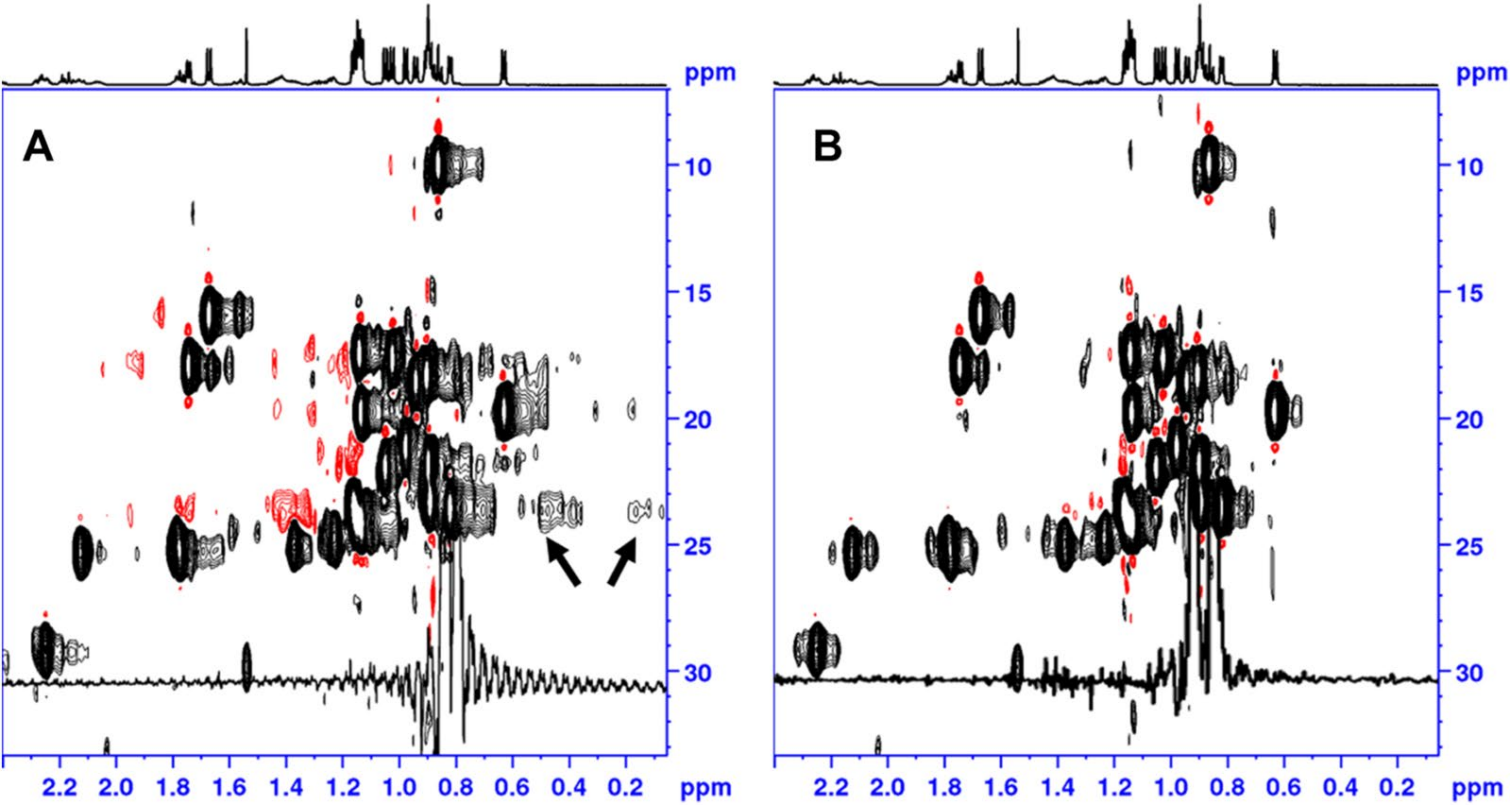
The spectra were collected using a 25 mM solution of Cyclosporine in deuterated benzene (C_6_D_6_) utilizing the pulse sequence depicted in Fig. 1. The BBHD HSQC spectrum in Fig. 1A was recorded using the hsqcetgpsp.2_bbhd pulse program as given in the Bruker library as reference for comparison. The reference experiment is using a BIRD^d^ pulse cascade and no variation of delay length to produce the spectrum. Sideband artefacts around the methyl region (δ^1^H/^13^C 0.8/23 ppm) are detected. The BIRD^r,X^ spectrum without RRD shows identical artefacts and is hence not shown. In (**B**) the applied RRD real time HD (BIRD^r,X^) delivers a better suppression of those sideband artefacts. During every scan, the length of data chunks as well as RF blocks is slightly changed between distinct previously set times. Yet the same delay settings are used from increment to increment. A significant reduction of signal sidebands is achieved leading to a better noise characteristic (see Fig. 3). The RRD real time BIRD pulse sequence (Fig. 1) is utilizing a set of limited randomly generated delays. The experiments in this paper were recorded using a Bruker-Avance III spectrometer (Bruker BioSpin, Rheinstetten, Germany) at 600.13 MHz proton frequency. The system is equipped with a 5 mm triple resonance TCI-Cryoprobe. A z-axis pulsed field gradient unit (PFG) was used either for coherence selection or as crusher gradients to destroy unwanted magnetization. The spectra shown in this paper were processed with the processing software Topspin 3.6.2^46^. The temperature for all samples was kept at 298 K. The experiment utilized a ^13^C sweep width of 22.64 kHz (t_2_(max) of 5.65 ms with an FID resolution of 176.8 Hz/point, number of complex points: 128). The spectral width in direct ^1^H dimension was set to 12,019.2 Hz giving an acquisition time of 127.7 ms with a physical dwell time of 83.2 us (number of complex points: 1536). All spectra were acquired using non-uniform sampling to speed up data acquisition. The built-in Topspin nussampler was used to create the point spread^47^. A NUS amount of 40% was chosen to sample the data points in the indirect dimension leading to 51 of 128 complex points. NUS reconstruction was done using compressed sensing (CS)^47–52^. Total measurement time of the reference and RRD real time homodecoupled HSQC experiment was 33 min. The 2D data set was apodised by multiplying with a shifted sine squared function (shift of 90°) and then zero-filled to yield a final matrix of 4096 (F_2_) × 1024 (F_1_) data points. In case a post processing phase correction in F_2_ is required, a Hilbert transform can be executed to retrieve the imaginary data points in F_2_, not calculated by NUS reconstruction (TopSpin command xht2)^46^. All spectra in Figs. 2, 4 and 5 were plotted by Topspin 3.6.2 and exported in TIFF format utilizing Microsoft PowerPoint for Microsoft 365 MSO (16.0.13801.20928) 64-bit graphic export filter.

The restricted random BIRD decoupling 2D HSQC sequence was initially applied to Cyclosporine to proof the reduction of sidebands in the 2D HSQC spectra. Figure 2 displays the comparison of the RRD (Fig. 2B, BIRD^r,X^) and conventional real time HD 2D HSQC spectra (Fig. 2A, BIRD^d^). The BIRD^r,X^ spectrum without RRD shows identical artefacts as 2A and is hence not shown.

In Fig. 2A (conventional) sideband artefacts around the methyl groups at ^1^H and ^13^C resonances (0.8/24 ppm) could be detected, displayed by arrows and insert. While in Fig. 2B (RRD), artefacts are reduced to the level of the noise as indicated in the 1D insert. The repetition of equally spaced delays was strictly avoided by randomly cycling all delay lengths within the real time HD block. However, this delay variation was constrained to a limited number of randomly chosen delays. A tradeoff between the magnitude of sideband suppression in ω_2_ and reduction of t_1_ noise has to be made. A good compromise was found to efficiently suppress the sideband artefacts in ω_2_ without a significant increase in t_1_ noise.

**Figure 3:**
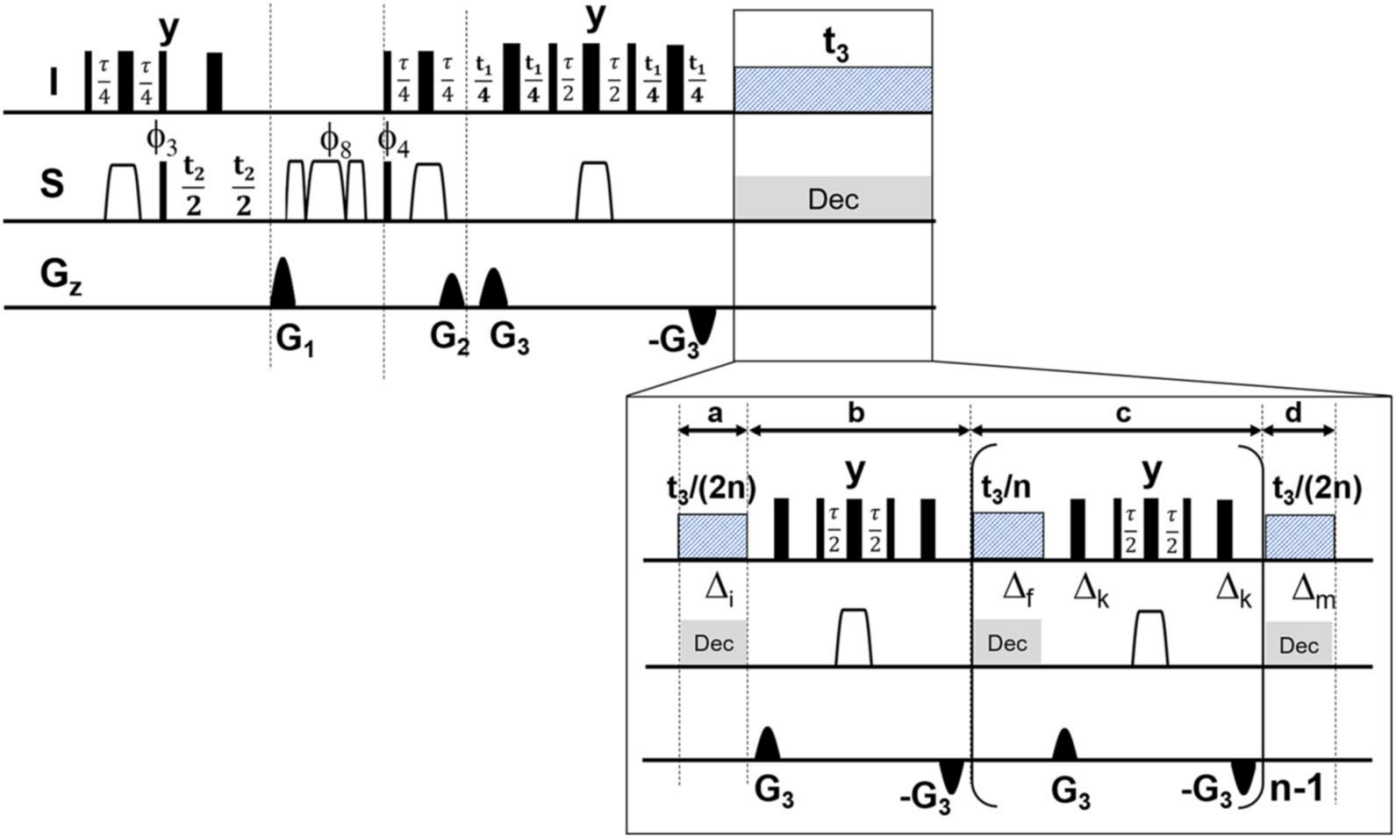
For obtaining the complete reduction of all proton spin multiplicities in multidimensional 2D NMR spectrum, the following 3D pulse sequence is used. The depicted pulse sequence is using the same RRD real time building block for decoupling in the observe dimension. A JRES element is introduced as third dimension to refocus the doublet splitting from diastereotopic CH_2_ groups which are unaffected by the BIRD elements in the observe dimension in t_3_. Unless otherwise stated almost all parameters related to the 2D HSQC direct detection remained unchanged and can be taken from Fig. 1. A delay compensated BIRDr,X was used to evolve the germinal ^2^J_HH_ homonuclear coupling in the indirect t_1_ dimension. Phase sensitive JRES in the indirect dimension is acquired using echo processing^19–22,36^.

After implementing the RRD element with reduced sideband artefacts, we turned our attention to solve the problem of residual doublet splitting of diastereotopic CH_2_ groups not removable by BIRD decoupling. To obtain a fully homodecoupled HSQC spectrum with final removal of diastereotopic doublet splitting, we combined two concepts into a 3D experiment. The combination of the RRD HD with phase sensitive JRES in an extra dimension directly provided a fully homodecoupled HSQC spectrum with all types of proton spin multiplicities removed. A long-sought way for complete reduction of all spin multiplicities with no restrictions is found. The resulting 3D pulse sequence is given in Fig. 3.

During the incrementation in the t_1_ dimension (JRES) only the scalar geminal ^2^J_HH_ coupling of the residual doublet splitting is left, because the JRES part uses the same modified BIRD^r,X^ block. This still generates a typical tilt in the spectrum. Because of the exclusive ^2^J_HH_ evolution only few data points in the JRES dimension must be recorded, regardless of the proton spin system complexity. No prior knowledge is required to perform the experiment.

**Figure 4:**
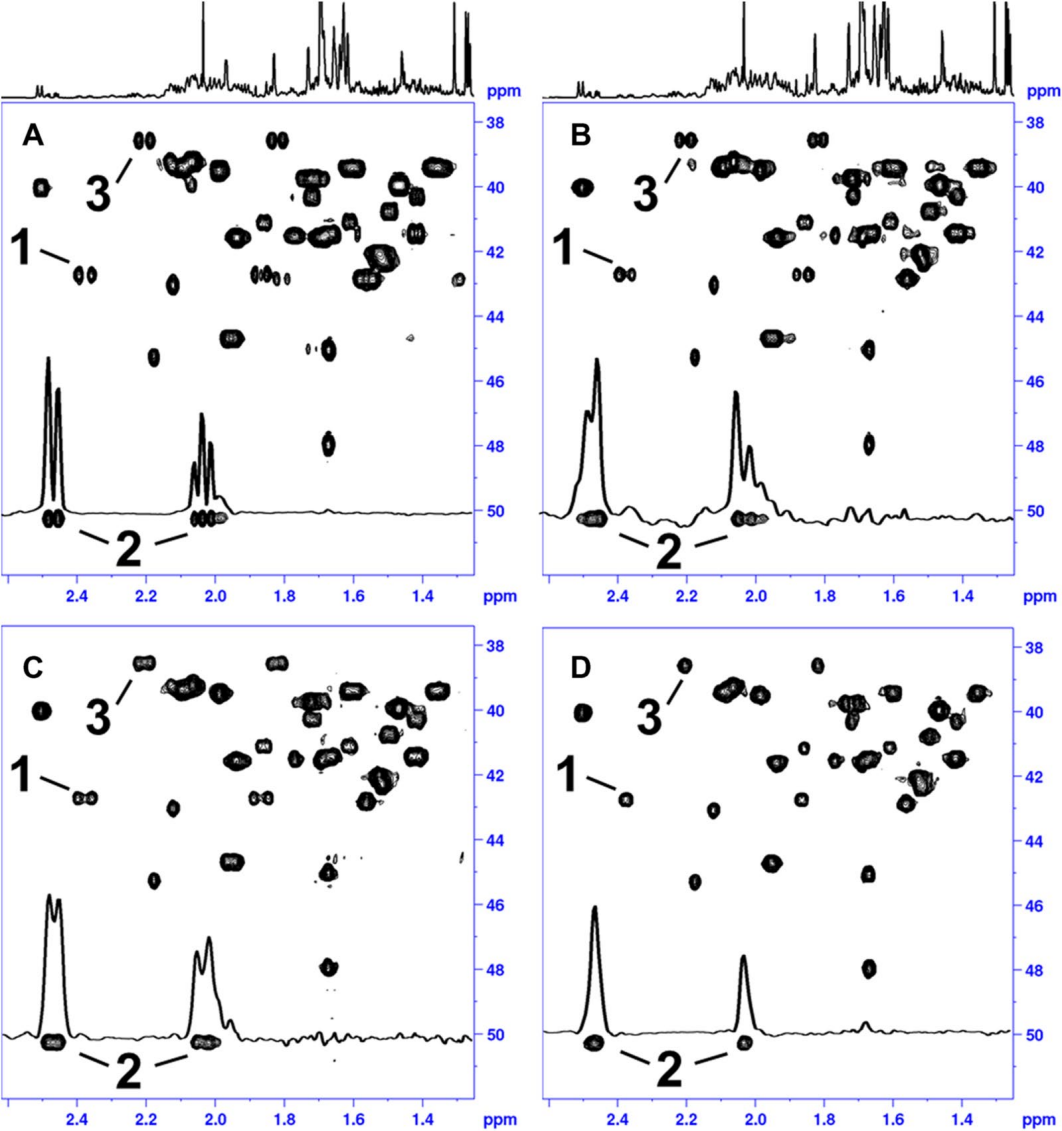
The spectra were collected on a Cannabis Terpene Mix solution in deuterated methanol. The sample was purchased from MERCK SUPELCO as a certified reference material, 2000 μg/mL each component in methanol (TraceCERT). The original Terpene TraceCERT solution was treated with a stream of nitrogen at room temperature to remove the initial methanol solvent and redissolved in Methanol-d_4_ prior to be used. The Terpene mix consists of following single Terpene components: beta-Pinene, Camphene, alpha-Pinene, 3-Carene, alpha-Terpinene, (R)-(+)-Limonene, gamma-Terpinene, L-(−)-Fenchone, Fenchol, (1R)-(+)-Camphor, Isoborneol, Menthol, Citronellol, (+)-Pulegone, Geranyl acetate, alpha-Cedrene, alpha-Humulene, Nerolidol, (+)-Cedrol, (−)-alpha Bisabolol. This figure displays an expansion of the spectra of the sample mixture. In (**A**) a standard HSQC with no homodecoupling is shown, (**B**) displays the literature known HD HSQC with BIRD^d^ element using the pulse sequence hsqcetgpsp.2_bbhd (Bruker release version). All 2D HSQC spectra A-C were recorded using NUS employing following parameters: A NUSamount of 40% was chosen to sample the data points in the indirect dimension leading to 204 of 512 complex points. NUS reconstruction was done using compressed sensing (CS)^47–52^. In (**C**) the result of restricted random HD 2D HSQC as described in text is presented and (**D**) shows the fully homodecoupled HSQC spectrum, where all multiplicities are reduced to singlet response. For the 3D experiment the spectral width in direct ^1^H dimension was 12,019.2 Hz leading to an acquisition time of 127.7 ms with a physical dwell time of 83.2 us (number of complex points: 1536). The experiment used a ^13^C sweep width of 22.64 kHz (t_2_(max) of 22.62 ms with an FID resolution of 44.2 Hz/point, number of complex points: 512). The ^1^H JRES dimension used a sweep width of 100 Hz (t_1_(max) of 160 ms with an FID resolution of 6.25 Hz/point, number of real points 16). The 3D experiment was acquired using non-uniform sampling to speed up data acquisition. The built-in Topspin nussampler was used to create the point spread^47^. A NUS amount of 8% was chosen to sample the data points in the indirect dimension leading to 655 of 8192 complex points. Exponential weighting is used to accommodate relaxation into the point spread (NusT_2_, ^13^C F_2_(20 m), ^1^H J res F_1_(150 m)). Total measurement time of the RRD real time BIRD homodecoupled 3D HSQC experiment was 8 h 8 min. The experiment could be run with 4 transients reducing the total measurement time by half with no significant loss of spectral quality. After FT in t_3_ and the reconstruction of the NUS data is done using compressed sensing (CS)^47–52^ and the processing is continued in the following order: FT in ^13^C dimension (t_2_), JRES dimension (t_1_). In the observe and ^13^C dimension data were multiplied by a shifted sine squared function (shift of 90°) and zero filled. The FIDs in the JRES dimension F_1_ were right shifted by 16 data points, linear backward predicted to give a total number of 32 points, multiplied with an unshifted sine window function and phase corrected using a large first order phase correction (PHC1 = 360° × 16 points = 5760°) to yield a final matrix of 4096 (F_3_) × 1024 (F_2_) × 32 (F_1_) data points. After that, a regular tilt processing of the F_1_F_3_ plane (J resolved dimension) for each F_2_ (^13^C) data point is carried out. The final fully broadband HD 2D HSQC spectrum is obtained by calculating a F_2_F_3_ projection along the number of F_1_ (^1^H) data points. In case a post processing phase correction in F2 is required, a Hilbert transform can be executed to retrieve the imaginary data points in F_2_, not calculated by NUS reconstruction (TopSpin command xht2)^46^.

**Figure 5:**
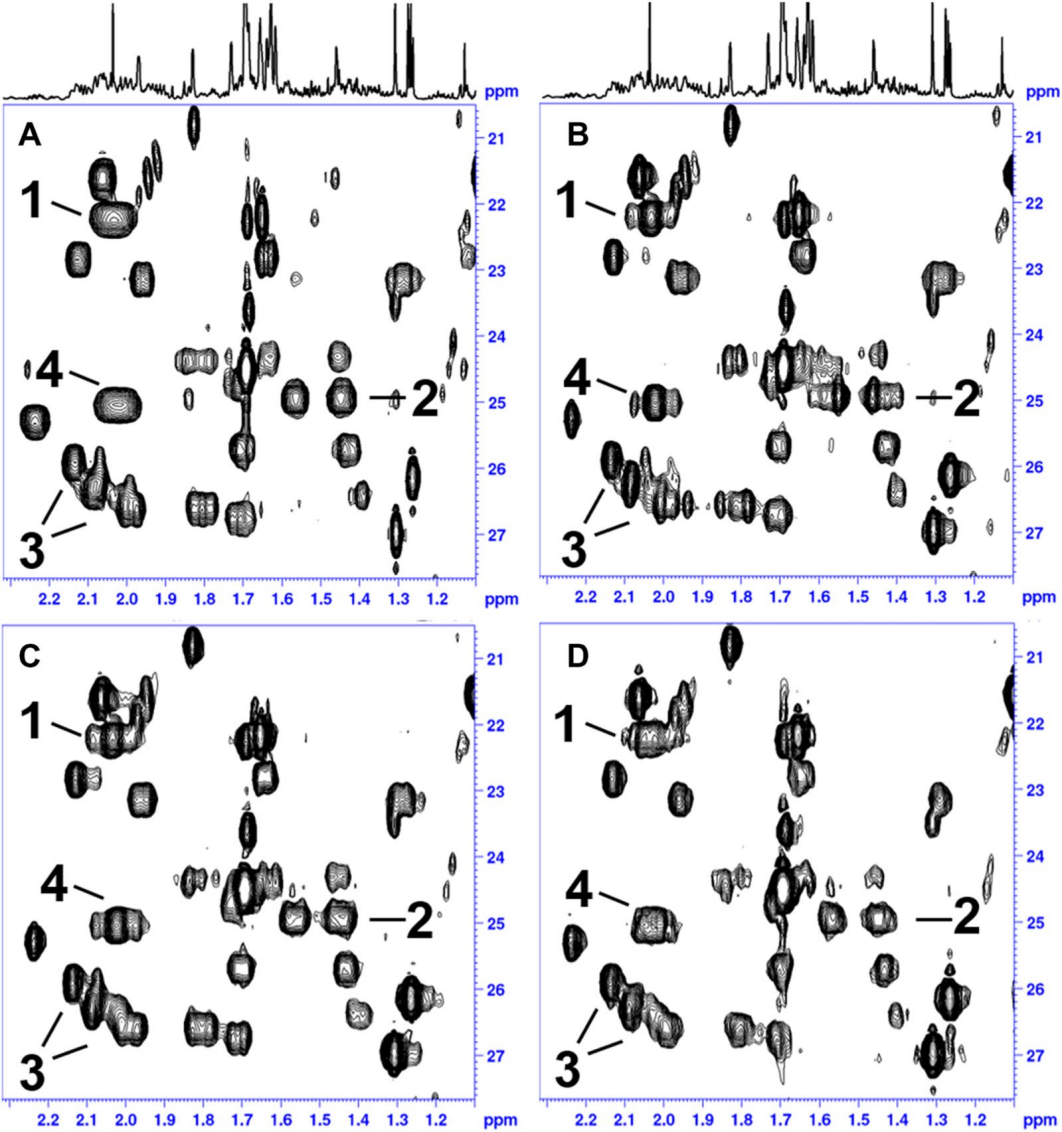
The spectra were collected on a Cannabis Terpene Mix solution in deuterated methanol. Detailed information on sample composition and experimental parameters is given in figure caption 4. This figure  presents additional expansions of the sample mixture, in (**A**) a standard HSQC with no homodecoupling is shown, (**B**) displays the literature known HD HSQC with BIRD^d^ element using the pulse sequence hsqcetgpsp.2_bbhd (Bruker release version), in (**C**) the result of restricted random HD 2D HSQC as described in text is presented and (**D**) shows the fully homodecoupled HSQC spectrum, where all multiplicities are reduced to singlet response. Diminished sidebands are clearly visible for the peaks annotated as 2 and 3. In (**B**), peaks denoted as 3 show significant sidebands in the 2D HD spectrum. While significant removal of sidebands is displayed in (**C**,**D**).

The RRD 3D experiment in Fig. 3 was recorded with NUS data sampling to significantly reduce the total measurement time while just 16 data points in the JRES dimension were sufficient to resolve the geminal ^2^J_HH_ doublet splitting in the F_1_ dimension. After FT and NUS reconstruction, the JRES dimension was processed by echo processing^19–22,36^ to deliver a fully phase sensitive 3D spectrum. As described earlier, every F_1_F_3_ plane was tilted and the F_2_F_3_ projection was calculated to produce the complete HD 2D HSQC spectrum (Figs. 4D, 5D). Figures 4 and 5 illustrate the results of the different experiments acquired using a Terpene mixture. Figures 4A and 5A show the standard HSQC. To observe the performance of HD among the different approaches, 1D ω_2_ traces at δ^13^C chemical shift of ca. 50 ppm were extracted (Fig. 4). We chose these 1D ω_2_ traces, because the corresponding ^13^C signal in F_1_ belongs to a diastereotopic CH_2_ group which is immune to HD and leaves an irreducible doublet splitting after HD. In case of the literature known HD sequence employing BIRD^d^ filter, a reduction of proton spin multiplicities was evident (Figs. 4B, 5B). However, some sidebands due to chunk data acquisition could still be detected compromising a possible signal assignment. The combination of restricted random delay (RRD) and BIRD^r,X^ filter was found to be reasonable for the suppression of signal sidebands in 2D HD spectra (Figs. 4C, 5C). On top of that, the RRD approach seems to give a better (less distorted) lineshape after HD as evidenced by comparison of ω_2_-traces in Fig. 4B,C. Figures 4D and 5D display the fully broadband homodecoupled spectrum with significant resolution enhancement compared to conventional HSQC spectrum (Figs. 4A, 5A). As indicated in Fig. 4 (peak 1–4) the doublet splitting from CH_2_ groups is clearly reduced to singlet responses removing the ambiguity of signal assignment in complex mixtures. The linewidth of the final proton signal is depending on at least two contributions. The first is the quality of the decoupling as such, which should have accomplished a complete reduction of all multiplets to singlets and at the same time a minimum level of signal sideband artefacts. The second important contribution is the proton T_2_ relaxation of the spins of the given sample, which will, given proper decoupling, eventually dominate the linewidth. This is especially evident for samples with short T_2_ values like proteins. For the current sample this becomes obvious when comparing the 1D trace of Fig. 4A,C, where in the latter spectrum the splitting is reduced. This goes along with a broadening of the lines. Similarly, the line in Fig. 4D is broader than the one in 4A. Yet, this is more than sufficient remove overlap and allows an unambiguous peak picking.

The RRD technique is expected to find widespread application in other HD experiments and in ZS based real time approaches to significantly reduce the occurrence of sidebands. Works along these lines are underway.

## Conclusion

Two major technical improvements on multidimensional HD HSQC NMR spectra are reported. In a first step, we have significantly diminished the intensity of the signal sidebands usually present in conventional HD HSQC spectra. This step has already made a straightforward signal assignment much easier and more reliable. This goal was achieved by variation of the RF and chunk block lengths during the physical acquisition. The block lengths were altered using a restricted number of randomly chosen values. By this, we avoided an elevated t_1_ noise which would otherwise show up in case of a fully random variation. Secondly, the obstacle of irreducible doublet splitting from diastereotopic CH_2_ groups was solved. A simple JRES element using a proton chemical shift compensated BIRD^r,X^ block was prepended to the sideband reduced HD HSQC experiment. The resulting 3D experiment was run with only 16 data points in JRES dimension to fully propagate the residual ^2^J_HH_ coupling. Finally, a phase sensitive echo processing and tilting along the JRES direction and projection delivered the fully multiplet free HD HSQC spectrum.

## Supplementary Information

Supplementary Information.

## Data Availability

The datasets generated and/or analyzed during the current study are available from the corresponding author upon request. Pulse programs used to produce the RRD HD HSQC spectra can be found in the supplementary material section.

## References

[1] Braun S, Kalinowski HO, Berger S (1998). 150 and More Basic NMR Experiments, A Practical Course 2nd Expanded Edition.

[2] Griesinger C, Schwalbe H, Schleucher J, Sattler M, Croasmun WR, Carlson RMK (1994). Proton-Detected Heteronuclear and Multidimensional NMR, Chapter 3, Two-Dimensional NMR Spectroscopy, Application for Chemists and Biochemists.

[3] Palmer AG, Cavanagh J, Wright PE, Rance M (1991). Sensitivity improvement in proton-detected two-dimensional heteronuclear correlation NMR spectroscopy. J. Magn. Reson..

[4] Kay LE, Keifer P, Saarinen T (1992). Pure absorption gradient enhanced heteronuclear single quantum correlation spectroscopy with improved sensitivity. J. Am. Chem. Soc..

[5] Schleucher J, Schwendinger M, Sattler M, Schmidt P, Schedletzky O, Glaser SJ, Sorensen OW, Griesinger C (1994). A general enhancement scheme in heteronuclear multidimensional NMR employing pulsed field gradients. J. Biomol. NMR.

[6] Wilker W, Leibfritz D, Kerssebaum R, Bermel W (1993). Gradient selection in inverse heteronuclear correlation spectroscopy. Magn. Reson. Chem..

[7] Zwahlen C, Legault P, Vincent SJF, Greenblatt J, Konrat R, Kay LE (1997). Methods for measurement of intermolecular NOEs by multinuclear NMR spectroscopy: Application to a bacteriophage N-Peptide/boxB RNA complex. J. Am. Chem. Soc..

[8] Carnevale D, Segawa TF, Bodenhausen G (2012). Polychromatic decoupling of a manifold of homonuclear scalar interactions in solution-State NMR. Chem. Eur. J..

[9] Garbow JR, Weitekamp DP, Pines A (1982). Bilinear rotation decoupling of homonuclear scalar interactions. Chem. Phys. Lett..

[10] Zangger K, Sterk H (1997). Homonuclear broadband-decoupled NMR spectra. J. Magn. Reson..

[11] Zangger K (2015). Pure shift NMR. Progr. NMR Spectr..

[12] Lupulescu A, Olsen GL, Frydman L (2012). Toward single-shot pure-shift solution 1H NMR by trains of BIRD-based homonuclear decoupling. J. Magn. Reson..

[13] Sakhaii P, Haase B, Bermel W (2009). Experimental access to HSQC spectra decoupled in all frequency dimensions. J. Magn. Reson..

[14] Aue WP, Karhan J, Ernst RR (1976). Homonuclear broadband decoupling and two-dimensional J-resolved NMR spectroscopy. J. Chem. Phys..

[15] Nagayama K, Bachmann P, Wuethrich K, Ernst RR (1978). The use of cross-sections and projections in two-dimensional NMR spectroscopy. J. Magn. Reson..

[16] Hahn EL (1950). Spin echos. Phys. Rev..

[17] Hahn EL, Maxwell DE (1952). Spin echo measurements of nuclear spin coupling in molecules. Phys. Rev..

[18] Segawa TF, Bodenhausen G (2013). Modulations of spin echos in liquids. eMagRes.

[19] Sakhaii P, Bermel W (2014). Improving the sensitivity of conventional spin echo spectra by preservation of initial signal-to-noise ratio. J. Magn. Reson..

[20] Nuzillard JM (1996). Time-reversal of NMR signals by linear prediction. Application to phase-sensitive homonuclear J-resolved spectroscopy. J. Magn. Reson. A.

[21] Marshall AG, Verdun FR (1990). ‘Fourier Transforms in NMR, Optical, and Mass Spectrometry.

[22] Martinez A, Bourdreux F, Riguet E, Nuzillard JM (2012). High-resolution and high sensitivity 2D homonuclear J-resolved spectroscopy. Magn. Reson. Chem..

[23] Pell AJ, Edden RAE, Keeler J (2007). Broadband proton-decoupled proton spectra. Magn. Reson. Chem..

[24] Foroozandeh M, Adams RW, Meharry NJ, Jeannerat D, Nilsson M, Morris GA (2014). Ultrahigh-resolution NMR spectroscopy. Angew. Chem. Int. Ed..

[25] Meyer NH, Zangger K (2013). Simplifying proton NMR spectra by instant homonuclear broadband decoupling. Angew. Chem. Int. Ed..

[26] Paudel L, Adams RW, Kiraly P, Aguilar JA, Foroozandeh M, Cliff MJ, Nilsson M, Sandor P, Waltho JP, Morris GA (2013). simultaneously enhancing spectral resolution and sensitivity in heteronuclear correlation NMR spectroscopy. Angew. Chem. Int. Ed..

[27] Ying J, Roche J, Bax A (2014). Homonuclear decoupling for enhancing resolution and sensitivity in NOE and RDC measurements of peptides and proteins. J. Magn. Reson..

[28] Struppe JO, Yang C, Wang Y, Hernandez RV, Shamansky LM, Mueller LJ (2013). Long-observation-window band-selective homonuclear decoupling: Increased sensitivity and resolution in solid-state NMR spectroscopy of proteins. J Magn. Reson..

[29] Castañar L, Nolis P, Virgili A, Parella T (2013). Full sensitivity and enhanced resolution in homodecoupled band-selective NMR experiments. Chem. Eur. J..

[30] Uhrin D, Liptaj T, Koever K (1993). Modified BIRD pulses and design of heteronuclear pulse sequences. J. Magn. Reson. A.

[31] Aguilar JA, Nilsson M, Morris GA (2011). Simple proton spectra from complex spin systems: Pure shift NMR spectroscopy using BIRD. Angew. Chem..

[32] Kaltschnee L, Kolmer A, Timári I, Schmidts V, Adams RW, Nilsson M, Kövér KE, Morris GA, Thiele CM (2014). ‘‘Perfecting” pure shift HSQC: Full homodecoupling for accurate and precise determination of heteronuclear couplings. Chem. Commun..

[33] Castañar L, Parella T (2015). Broadband 1H homodecoupled NMR experiments: Recent developments, methods and applications. Magn. Reson. Chem..

[34] Li H, Yang Y, Zhan H, Lin X, Chen Z (2021). Periodic artifact suppression for pure shift NMR spectroscopy. IEEE Trans. Instrum. Meas..

[35] John BK, Plant D, Hurd RE (1993). Improved proton-detected heteronuclear correlation using gradient-enhanced Z and ZZ filters. J. Magn. Reson. A.

[36] Sakhaii P, Bohorc B, Bermel W (2018). Small angle double quantum spectroscopy (SAQS NMR). J. Magn. Reason..

[37] Gravina S, Cory DG (1994). Sensitivity and resolution of constant-time imaging. J. Magn. Reason. B.

[38] Meyer NH, Zangger K (2014). Enhancing the resolution of multi-dimensional heteronuclear NMR spectra of intrinsically disordered proteins by homonuclear broadband decoupling. Chem. Commun..

[39] Mauhart J, Glanzer S, Sakhaii P, Bermel W, Zangger K (2015). Faster and cleaner real-time pure shift NMR experiments. J. Magn. Reson..

[40] Sakhaii P, Haase B, Bermel W, Kerssebaum R, Wagner GE, Zangger K (2013). Broadband homodecoupled NMR spectroscopy with enhanced sensitivity. J. Magn. Reson..

[41] Wagner GE, Sakhaii P, Bermel W, Zangger K (2013). Monitoring fast reactions by spatially selective and frequency-shifted continuous NMR spectroscopy: Application to rapid-injection protein unfolding. Chem. Commun..

[42] Morris GA, Freeman R (1979). Enhancement of nuclear magnetic resonance signals by polarization transfer. J. Am. Chem. Soc..

[43] TOPSPIN 3.1 Patch Level 6: Chirp pulse Crp60comp.4 was taken from Standard Bruker Library.

[44] Fu R, Bodenhausen G (1995). Broadband decoupling in NMR with frequency-modulated chirp pulses. Chem. Phys. Lett..

[45] Shaka AJ, Barker PB, Freeman R (1985). Computer-optimized decoupling scheme for wideband applications and low-level operation. J. Magn. Reson..

[46] TOPSPIN 3.6.2 (of 2019-07-22 14:46:29) Service Pack: 0 GUI build number: 203 Java version: 1.8.0_202 (32 bit) Oracle Corporation. https://www.bruker.com/en/products-and-solutions/mr/nmr-software/topspin.html.

[47] Orekhov VY, Jaravine VA (2011). Analysis of non-uniformly sampled spectra with multi-dimensional decomposition. Prog. Nucl. Magn. Reson. Spectrosc..

[48] Donoho DL (2006). Compressed sensing. IEEE Trans Inf. Theory.

[49] Candes EJ, Romberg J, Tao T (2006). Robust uncertainty principles: Exact signal reconstruction from highly incomplete frequency information. IEEE Trans Inf. Theory.

[50] Drori, I. Fast l1 Minimization by iterative thresholding for multidimensional NMR spectroscopy. *EURASIP J. Adv. Sig. Pr.* Article ID 20248 (2007).

[51] Kazimierczuk K, Orekhov VY (2011). Accelerated NMR spectroscopy by using compressed sensing. Angew. Chem. Int. Ed..

[52] Holland DJ, Bostock MJ, Gladden LF, Nietlispach D (2011). Fast multidimensional NMR spectroscopy using compressed sensing. Angew. Chem. Int. Ed..

