# Supplementary material for: Boosting the resolution of multidimensional NMR spectra by complete removal of proton spin multiplicities
Source: Sci Rep. 2021 Nov 3;11:21566. doi: 10.1038/s41598-021-01041-8 (PMC8566458; doi:10.1038/s41598-021-01041-8)
Supplement: Supplementary file 1 — Supplementary Information. [file 41598_2021_1041_MOESM1_ESM.docx]

**Boosting the resolution of multidimensional NMR spectra by complete removal of proton spin multiplicities**

Peyman Sakhaii^1^*, Bojan Bohorc^1^, Uwe Schliedermann^1^ & Wolfgang Bermel^2^

Author information

Affiliations

1. **NMR Laboratory of Global CMC Early Development, Synthetics Platform, industrial park Hoechst, Building G849, D-65926 Frankfurt/Main, Germany**

Peyman Sakhaii (corresponding author), Bojan Bohorc & Uwe Schliedermann

1. **Bruker BioSpin GmbH, Silberstreifen 4, D-76287 Rheinstetten, Germany**

Wolfgang Bermel

Supplementary material

The supplementary material contains 2 pulse sequences used to acquire the Restricted Random Delay HD HSQC spectra. The Bruker pulse sequences are given in Bruker format suitable for AV III / HD electronic consoles. Both sequences are making use of the RRD to efficiently suppress the signal sidebands usually present in conventional HD spectra.

You agree that you are making use of the pulse sequences at your own risk, and that the sequences being provided to you on an “AS IS” and “AS AVAILABLE” basis. No warranty shall be accepted for improper use or wrong parameter settings of the delivered HD HSQC sequences.

Table caption S1

The table S1 displays the restricted random delays (RRD) delays used for diminishing the signal sidebands employed during the HD in *hsqcetgpsp.2_rrd2d* and *hsqcetgpsp.2_rrd3d* pulse sequences. As described in the text, a restricted number of randomly chosen delays was used. The delays a, b, c and d are annotated in the corresponding pulse sequences given in Figures 1 (*hsqcetgpsp.2_rrd2d*) and 3 (*hsqcetgpsp.2_rrd3d*). All 2D and 3D HD HSQC experiments were recorded using a loop parameter n of 12 (Figure 1 and 3).

The random numbers were generated using the comprehensive random generator machine given at [https://www.calculator.net/ random-number-generator.html](https://www.calculator.net/%20random-number-generator.html).

Table S1

|  | | LOOP | Number of scans | | | | | | | | | | | | | | | |
| --- | --- | --- | --- | --- | --- | --- | --- | --- | --- | --- | --- | --- | --- | --- | --- | --- | --- | --- |
| Delays [sec] | |  | 1 | 2 | 3 | 4 | 5 | 6 | 7 | 8 | 9 | 10 | 11 | 12 | 13 | 14 | 15 | 16 |
| a | |  | 0.00997 | 0.00153 | 0.00631 | 0.00044 | 0.00687 | 0.00808 | 0.00876 | 0.00562 | 0.00789 | 0.00655 | 0.0022 | 0.000508 | 0.00789 | 0.00808 | 0.00257 | 0.00044 |
| b | |  | 0 | 0 | 0 | 0 | 0 | 0 | 0 | 0 | 0 | 0 | 0 | 0 | 0 | 0 | 0 | 0 |
| c | Δfn | 1 | 0 | 0 | 0 | 0 | 0 | 0 | 0 | 0 | 0 | 0 | 0 | 0 | 0 | 0 | 0 | 0 |
|  |  | 2 | 0 | 0 | 0 | 0 | 0 | 0 | 0 | 0 | 0 | 0 | 0 | 0 | 0 | 0 | 0 | 0 |
|  |  | 3 | 0.00645 | 0.00645 | 0.00645 | 0.00645 | 0.00645 | 0.00645 | 0.00645 | 0.00645 | 0.00645 | 0.00645 | 0.00645 | 0.00645 | 0.00645 | 0.00645 | 0.00645 | 0.00645 |
|  |  | 4 | 0.00645 | 0.00645 | 0.00645 | 0.00645 | 0.00645 | 0.00645 | 0.00645 | 0.00645 | 0.00645 | 0.00645 | 0.00645 | 0.00645 | 0.00645 | 0.00645 | 0.00645 | 0.00645 |
|  |  | 5 | 0.00042 | 0.00042 | 0.00042 | 0.00042 | 0.00042 | 0.00042 | 0.00042 | 0.00042 | 0.00042 | 0.00042 | 0.00042 | 0.00042 | 0.00042 | 0.00042 | 0.00042 | 0.00042 |
|  |  | 6 | 0.00042 | 0.00042 | 0.00042 | 0.00042 | 0.00042 | 0.00042 | 0.00042 | 0.00042 | 0.00042 | 0.00042 | 0.00042 | 0.00042 | 0.00042 | 0.00042 | 0.00042 | 0.00042 |
|  |  | 7 | 0.00257 | 0.00257 | 0.00257 | 0.00257 | 0.00257 | 0.00257 | 0.00257 | 0.00257 | 0.00257 | 0.00257 | 0.00257 | 0.00257 | 0.00257 | 0.00257 | 0.00257 | 0.00257 |
|  |  | 8 | 0.00257 | 0.00257 | 0.00257 | 0.00257 | 0.00257 | 0.00257 | 0.00257 | 0.00257 | 0.00257 | 0.00257 | 0.00257 | 0.00257 | 0.00257 | 0.00257 | 0.00257 | 0.00257 |
|  |  | 9 | 0.00208 | 0.00208 | 0.00208 | 0.00208 | 0.00208 | 0.00208 | 0.00208 | 0.00208 | 0.00208 | 0.00208 | 0.00208 | 0.00208 | 0.00208 | 0.00208 | 0.00208 | 0.00208 |
|  |  | 10 | 0.00208 | 0.00208 | 0.00208 | 0.00208 | 0.00208 | 0.00208 | 0.00208 | 0.00208 | 0.00208 | 0.00208 | 0.00208 | 0.00208 | 0.00208 | 0.00208 | 0.00208 | 0.00208 |
|  |  | 11 | 0.00305 | 0.00305 | 0.00305 | 0.00305 | 0.00305 | 0.00305 | 0.00305 | 0.00305 | 0.00305 | 0.00305 | 0.00305 | 0.00305 | 0.00305 | 0.00305 | 0.00305 | 0.00305 |
|  |  | 12 | 0.00305 | 0.00305 | 0.00305 | 0.00305 | 0.00305 | 0.00305 | 0.00305 | 0.00305 | 0.00305 | 0.00305 | 0.00305 | 0.00305 | 0.00305 | 0.00305 | 0.00305 | 0.00305 |
| c | Δkn | 1 | 0.00344 | 0.00344 | 0.00344 | 0.00344 | 0.00344 | 0.00344 | 0.00344 | 0.00344 | 0.00344 | 0.00344 | 0.00344 | 0.00344 | 0.00344 | 0.00344 | 0.00344 | 0.00344 |
|  |  | 2 | 0.00404 | 0.00404 | 0.00404 | 0.00404 | 0.00404 | 0.00404 | 0.00404 | 0.00404 | 0.00404 | 0.00404 | 0.00404 | 0.00404 | 0.00404 | 0.00404 | 0.00404 | 0.00404 |
|  |  | 3 | 0.00058 | 0.00058 | 0.00058 | 0.00058 | 0.00058 | 0.00058 | 0.00058 | 0.00058 | 0.00058 | 0.00058 | 0.00058 | 0.00058 | 0.00058 | 0.00058 | 0.00058 | 0.00058 |
|  |  | 4 | 0.00166 | 0.00166 | 0.00166 | 0.00166 | 0.00166 | 0.00166 | 0.00166 | 0.00166 | 0.00166 | 0.00166 | 0.00166 | 0.00166 | 0.00166 | 0.00166 | 0.00166 | 0.00166 |
|  |  | 5 | 0.00498 | 0.00498 | 0.00498 | 0.00498 | 0.00498 | 0.00498 | 0.00498 | 0.00498 | 0.00498 | 0.00498 | 0.00498 | 0.00498 | 0.00498 | 0.00498 | 0.00498 | 0.00498 |
|  |  | 6 | 0.00076 | 0.00076 | 0.00076 | 0.00076 | 0.00076 | 0.00076 | 0.00076 | 0.00076 | 0.00076 | 0.00076 | 0.00076 | 0.00076 | 0.00076 | 0.00076 | 0.00076 | 0.00076 |
|  |  | 7 | 0.00316 | 0.00316 | 0.00316 | 0.00316 | 0.00316 | 0.00316 | 0.00316 | 0.00316 | 0.00316 | 0.00316 | 0.00316 | 0.00316 | 0.00316 | 0.00316 | 0.00316 | 0.00316 |
|  |  | 8 | 0.00022 | 0.00022 | 0.00022 | 0.00022 | 0.00022 | 0.00022 | 0.00022 | 0.00022 | 0.00022 | 0.00022 | 0.00022 | 0.00022 | 0.00022 | 0.00022 | 0.00022 | 0.00022 |
|  |  | 9 | 0.00394 | 0.00394 | 0.00394 | 0.00394 | 0.00394 | 0.00394 | 0.00394 | 0.00394 | 0.00394 | 0.00394 | 0.00394 | 0.00394 | 0.00394 | 0.00394 | 0.00394 | 0.00394 |
|  |  | 10 | 0.0012 | 0.0012 | 0.0012 | 0.0012 | 0.0012 | 0.0012 | 0.0012 | 0.0012 | 0.0012 | 0.0012 | 0.0012 | 0.0012 | 0.0012 | 0.0012 | 0.0012 | 0.0012 |
|  |  | 11 | 0.00226 | 0.00226 | 0.00226 | 0.00226 | 0.00226 | 0.00226 | 0.00226 | 0.00226 | 0.00226 | 0.00226 | 0.00226 | 0.00226 | 0.00226 | 0.00226 | 0.00226 | 0.00226 |
|  |  | 12 | 0.00454 | 0.00454 | 0.00454 | 0.00454 | 0.00454 | 0.00454 | 0.00454 | 0.00454 | 0.00454 | 0.00454 | 0.00454 | 0.00454 | 0.00454 | 0.00454 | 0.00454 | 0.00454 |
| d | |  | 0.00305 | 0.00876 | 0.00562 | 0.00687 | 0.00645 | 0.00042 | 0.00789 | 0.000257 | 0.00208 | 0.00542 | 0.00113 | 0.00227 | 0.00305 | 0.00687 | 0.00542 | 0.00645 |

Following pulse sequences are given in the following sections:

| Restricted random delay (RRD) 2D homodecoupled HSQC | *hsqcetgpsp.2_rrd2d* |
| --- | --- |
| JRES-RRD 3D homodecoupled HSQC (used to obtain full HD HSQC after TILT on the JRES dimension) | *hsqcetgpsp.2_rrd3d* |

;hsqcetgpsp.2_rrd2d

;06/04/2021 peyman sakhaii and wolfgang bermel

;2D H-1/X correlation via double inept transfer

;phase sensitive using Echo/Antiecho-TPPI gradient selection

;with decoupling during acquisition

;using trim pulses in inept transfer

;using shaped pulses for inversion and refocussing on f2 - channel

;using 1H broadband homodecoupling during acquisition with [BIRD]r,x element

;using dwellmode explicit

;CH2 groups may be left as doubletts with 2J(HH) spliting

; N.H. Meyer, K. Zangger, Simplifying proton NMR spectra by instant homonuclear ;broadband decoupling, Angew. Chem. Int. Ed. 52 (2013) 7143–7146.

;L. Paudel, R.W. Adams, P. Kiraly, J.A. Aguilar, M. Foroozandeh,

; M.J. Cliff, M. Nilsson, P. Sandor, J.P. Waltho & G.A. Morris,

; Angew. Chem. 125, 11830-11833 (2013)

;(J.A. Aguilar, M. Nilsson & G.A. Morris, Angew. Chem. 123, 9716-9717 (2011))

;(P. Sakhaii, B. Haase & W. Bermel, J. Magn. Reson. 199, 192-198 (2009))

;

;$CLASS=HighRes

;$DIM=2D

;$TYPE=

;$SUBTYPE=

;$COMMENT=

#include <Avance.incl>

#include <Grad.incl>

#include <Delay.incl>

#include <De.incl>

define list<delay> LIST1 = { 0 0 0.00645 0.00645 0.00042 0.00042 0.00257 0.00257 0.00208 0.00208 0.00305 0.00305 }

define list<delay> LIST2 = {0.00172 0.00202 0.00029 0.00083 0.00249 0.00038 0.00158 0.00011 0.00197 0.00060 0.00113 0.00227 }

define list<delay> LIST3 = { 0.00997 0.00153 0.00631 0.00044 0.00687 0.00808 0.00876 0.00562 0.00789 0.00655 0.00220 0.00508 0.00789 0.00808 0.00257 0.00044 }

define list<delay> LIST4 = { 0.00305 0.00876 0.00562 0.00687 0.00645 0.00042 0.00789 0.00257 0.00208 0.00542 0.00113 0.00227 0.00305 0.00687 0.00542 0.00645 }

"p2=p1*2"

"d2=1s/(cnst2*2)"

"d4=1s/(cnst2*4)"

"d11=30m"

"p29=300u"

# ifdef LABEL_CN

"p22=p21*2"

# else

# endif /*LABEL_CN*/

"d0=3u"

"in0=inf1/2"

"d62=aq/l0"

"d63=d62/2"

"l1=l0-1"

"DELTA1=d4-p16-d16-larger(p2,p14)/2-de-12u"

"DELTA2=d4-larger(p2,p14)/2"

"DELTA3=d4-larger(p2,p14)/2-p1*2/PI"

"DELTA4=d2-larger(p2,p39)/2"

"DELTA5=4u+p29+d16"

# ifdef LABEL_CN

"DELTA=p16+d16+larger(p2,p22)+d0*2"

# else

"DELTA=p16+d16+p2+d0*2"

# endif /*LABEL_CN*/

"acqt0=0"

baseopt_echo

dwellmode explicit

1 ze

d11 pl12:f2

2 d11 do:f2

4u BLKGRAD

d1 pl1:f1

50u UNBLKGRAD

3 (p1 ph1)

DELTA2

4u

(center (p2 ph1) (p14:sp3 ph6):f2 )

4u

DELTA2 pl2:f2

p28 ph1

4u

(p1 ph2) (p3 ph3):f2

d0

# ifdef LABEL_CN

(center (p2 ph5) (p22 ph1):f3 )

# else

(p2 ph5)

# endif /*LABEL_CN*/

d0

p16:gp1*EA

d16

4u

(p24:sp7 ph8):f2

4u

DELTA pl2:f2

(ralign (p1 ph1) (p3 ph4):f2 )

DELTA3 pl0:f2

(center (p2 ph1) (p14:sp3 ph1):f2 )

4u

p16:gp2

d16

DELTA1 pl12:f2

4u cpd2:f2

ACQ_START(ph30,ph31)

0.1u REC_UNBLK

0.05u DWL_CLK_ON

d63

LIST3 LIST3.inc

0.05u DWL_CLK_OFF

0.1u REC_BLK

4u do:f2

p29:gp3

d16 pl1:f1

(p2 ph1):f1

DELTA5

(p1 ph1)

DELTA4 pl0:f2

(center (p2 ph2) (p39:sp4 ph1):f2 )

DELTA4

(p1 ph1)

DELTA5

(p2 ph1):f1

4u pl12:f2

p29:gp3*-1

d16 cpd2:f2

;

4 0.1u REC_UNBLK

0.05u DWL_CLK_ON

d62

LIST1 LIST1.inc

0.05u DWL_CLK_OFF

0.1u REC_BLK

;

LIST2

4u do:f2

p29:gp3

d16 pl1:f1

(p2 ph1):f1

DELTA5

LIST2

(p1 ph1)

DELTA4 pl0:f2

(center (p2 ph2) (p39:sp4 ph1):f2 )

DELTA4

(p1 ph1)

DELTA5

LIST2

(p2 ph1):f1

4u pl12:f2

LIST2

p29:gp3*-1

d16 cpd2:f2 LIST2.inc

lo to 4 times l1

0.1u REC_UNBLK

0.05u DWL_CLK_ON

d62*2

LIST4 LIST4.inc

0.05u DWL_CLK_OFF

0.1u REC_BLK

rcyc=2

d11 do:f2 mc #0 to 2

F1EA(calgrad(EA), caldel(d0, +in0) & calph(ph3, +180) & calph(ph6, +180) & calph(ph31, +180))

4u BLKGRAD

exit

ph1=0

ph2=1

ph3=0 2

ph4=0 0 2 2

ph5=0

ph6=0

ph7=2

ph8=0 0 0 0 1 1 1 1

ph30=0

ph31=0 2 2 0 2 0 0 2

;pl1 : f1 channel - power level for pulse (default)

;pl2 : f2 channel - power level for pulse (default)

;pl3 : f3 channel - power level for pulse (default)

;pl12: f2 channel - power level for CPD/BB decoupling

;sp3: f2 channel - shaped pulse 180 degree for inversion

;spnam3: Crp60,0.5,20.1 (Crp80,0.5,20.1)

;sp4: f2 channel - shaped pulse (180degree refocussing)

;spnam4: Bip720,100,10.1

;sp7: f2 channel - shaped pulse 180 degree for refocussing

;spnam7: Crp60comp.4 (Crp80comp.4)

;p1 : f1 channel - 90 degree high power pulse

;p2 : f1 channel - 180 degree high power pulse

;p3 : f2 channel - 90 degree high power pulse

;p14: f2 channel - 180 degree shaped pulse for inversion

;p16: homospoil/gradient pulse

;p21: f3 channel - 90 degree high power pulse

;p22: f3 channel - 180 degree high power pulse

;p24: f2 channel - 180 degree shaped pulse for refocussing

;p28: f1 channel - trim pulse

;p29: gradient pulse 3 [300 usec]

;p39: f2 channel - 180 degree shaped pulse for refocussing

; Bip720,100,10.1 (160us at 600.13 MHz)

;d0 : incremented delay (2D) [3 usec]

;d1 : relaxation delay; 1-5 * T1

;d2 : 1/(2J)XH

;d4 : 1/(4J)XH

;d11: delay for disk I/O [30 msec]

;d16: delay for homospoil/gradient recovery

;d62: length of block between decoupling pulses : = aq/l0 [< 20-25 msec]

;d63: = d62/2

;cnst2: = J(XH)

;l0 : number of blocks during acquisition time

; adjust to get d62 as required

;inf1: 1/SW(X) = 2 * DW(X)

;in0: 1/(2 * SW(X)) = DW(X)

;nd0: 2

;ns: 1 * n

;ds: >= 16

;td1: number of experiments

;FnMODE: echo-antiecho

;cpd2: decoupling according to sequence defined by cpdprg2

;pcpd2: f2 channel - 90 degree pulse for decoupling sequence

;for z-only gradients:

;gpz1: 80%

;gpz2: 20.1% for C-13

;gpz3: 27%

;use gradient files:

;gpnam1: SMSQ10.100

;gpnam2: SMSQ10.100

;gpnam3: SMSQ10.50

;gpnam4: SMSQ10.50

;preprocessor-flags-start

;LABEL_CN: for C-13 and N-15 labeled samples start experiment with

; option -DLABEL_CN (eda: ZGOPTNS)

;preprocessor-flags-end

;$Id: $

;hsqcetgpsp.2_rrd3d

;restricted random delay bird decoupling and nus with JRES as 3D

;06/04/2021 peyman sakhaii and wolfgang bermel

;avance-version (22/04/2021)

;reset-HSQC

;2D H-1/X correlation via double inept transfer

;phase sensitive using Echo/Antiecho-TPPI gradient selection

;with decoupling during acquisition

;using trim pulses in inept transfer

;using shaped pulses for inversion and refocussing on f2 - channel

;using 1H broadband homodecoupling during acquisition with [BIRD]r,x element

;using dwellmode explicit

;CH2 groups may be left as doubletts with 2J(HH) spliting

; N.H. Meyer, K. Zangger, Simplifying proton NMR spectra by instant homonuclear ;broadband decoupling, Angew. Chem. Int. Ed. 52 (2013) 7143–7146.

;L. Paudel, R.W. Adams, P. Kiraly, J.A. Aguilar, M. Foroozandeh,

; M.J. Cliff, M. Nilsson, P. Sandor, J.P. Waltho & G.A. Morris,

; Angew. Chem. 125, 11830-11833 (2013)

;(J.A. Aguilar, M. Nilsson & G.A. Morris, Angew. Chem. 123, 9716-9717 (2011))

;(P. Sakhaii, B. Haase & W. Bermel, J. Magn. Reson. 199, 192-198 (2009))

;

;$CLASS=HighRes

;$DIM=2D

;$TYPE=

;$SUBTYPE=

;$COMMENT=

#include <Avance.incl>

#include <Grad.incl>

#include <Delay.incl>

#include <De.incl>

define list<delay> LIST1 = { 0 0 0.00645 0.00645 0.00042 0.00042 0.00257 0.00257 0.00208 0.00208 0.00305 0.00305 }

define list<delay> LIST2 = {0.00172 0.00202 0.00029 0.00083 0.00249 0.00038 0.00158 0.00011 0.00197 0.00060 0.00113 0.00227 }

define list<delay> LIST3 = { 0.00997 0.00153 0.00631 0.00044 0.00687 0.00808 0.00876 0.00562 0.00789 0.00655 0.00220 0.00508 0.00789 0.00808 0.00257 0.00044 }

define list<delay> LIST4 = { 0.00305 0.00876 0.00562 0.00687 0.00645 0.00042 0.00789 0.00257 0.00208 0.00542 0.00113 0.00227 0.00305 0.00687 0.00542 0.00645 }

"p2=p1*2"

"d2=1s/(cnst2*2)"

"d4=1s/(cnst2*4)"

"d11=30m"

"p29=300u"

# ifdef LABEL_CN

"p22=p21*2"

# else

# endif /*LABEL_CN*/

"d0=3u"

"d10=3u"

"in0=inf1/4"

"in10=inf2/2"

"d62=aq/l0"

"d63=d62/2"

"l1=l0-1"

"DELTA1=d4-p16-d16-larger(p2,p14)/2-de-12u"

"DELTA2=d4-larger(p2,p14)/2"

"DELTA3=d4-larger(p2,p14)/2-p1*2/PI"

"DELTA4=d2-larger(p2,p39)/2"

"DELTA5=4u+p29+d16"

# ifdef LABEL_CN

"DELTA=p16+d16+larger(p2,p22)+d0*2"

# else

"DELTA=p16+d16+p2+d0*2"

# endif /*LABEL_CN*/

"acqt0=0"

baseopt_echo

dwellmode explicit

aqseq 312

1 ze

d11 pl12:f2

2 d11 do:f2

4u BLKGRAD

d1 pl1:f1

50u UNBLKGRAD

3 (p1 ph1)

DELTA2

4u

(center (p2 ph1) (p14:sp3 ph6):f2 )

4u

DELTA2 pl2:f2

p28 ph1

4u

(p1 ph2) (p3 ph3):f2

d10

# ifdef LABEL_CN

(center (p2 ph5) (p22 ph1):f3 )

# else

(p2 ph5)

# endif /*LABEL_CN*/

d10

p16:gp1*EA

d16

4u

(p24:sp7 ph8):f2

4u

DELTA pl2:f2

(ralign (p1 ph1) (p3 ph4):f2 )

DELTA3 pl0:f2

(center (p2 ph1) (p14:sp3 ph1):f2 )

4u

p16:gp2

d16

DELTA1

; BIRDrx J res

d0

4u

p29:gp5

d16 pl1:f1

(p2 ph1):f1

d0

DELTA5

(p1 ph1)

DELTA4 pl0:f2

(center (p2 ph2) (p39:sp4 ph1):f2 )

DELTA4

(p1 ph1)

DELTA5

d0

(p2 ph1):f1

4u

p29:gp5*-1

d16

d0

; BIRDrx J res

4u pl12:f2

4u cpd2:f2

ACQ_START(ph30,ph31)

0.1u REC_UNBLK

0.05u DWL_CLK_ON

d63

LIST3 LIST3.inc

0.05u DWL_CLK_OFF

0.1u REC_BLK

4u do:f2

p29:gp3

d16 pl1:f1

(p2 ph1):f1

DELTA5

(p1 ph1)

DELTA4 pl0:f2

(center (p2 ph2) (p39:sp4 ph1):f2 )

DELTA4

(p1 ph1)

DELTA5

(p2 ph1):f1

4u pl12:f2

p29:gp3*-1

d16 cpd2:f2

;

4 0.1u REC_UNBLK

0.05u DWL_CLK_ON

d62

LIST1 LIST1.inc

0.05u DWL_CLK_OFF

0.1u REC_BLK

;

LIST2

4u do:f2

p29:gp3

d16 pl1:f1

(p2 ph1):f1

DELTA5

LIST2

(p1 ph1)

DELTA4 pl0:f2

(center (p2 ph2) (p39:sp4 ph1):f2 )

DELTA4

(p1 ph1)

DELTA5

LIST2

(p2 ph1):f1

4u pl12:f2

LIST2

p29:gp3*-1

d16 cpd2:f2 LIST2.inc

lo to 4 times l1

0.1u REC_UNBLK

0.05u DWL_CLK_ON

d62*2

LIST4 LIST4.inc

0.05u DWL_CLK_OFF

0.1u REC_BLK

rcyc=2

d11 do:f2 mc #0 to 2

F1QF(caldel(d0, +in0))

F2EA(calgrad(EA), caldel(d10, +in10) & calph(ph3, +180) & calph(ph6, +180) & calph(ph31, +180))

4u BLKGRAD

exit

ph1=0

ph2=1

ph3=0 2

ph4=0

ph5=0

ph6=0

ph7=2

ph8=0 0 1 1

ph30=0

ph31=0 2 2 0

;pl1 : f1 channel - power level for pulse (default)

;pl2 : f2 channel - power level for pulse (default)

;pl3 : f3 channel - power level for pulse (default)

;pl12: f2 channel - power level for CPD/BB decoupling

;sp3: f2 channel - shaped pulse 180 degree for inversion

;spnam3: Crp60,0.5,20.1 (Crp80,0.5,20.1)

;sp4: f2 channel - shaped pulse (180degree refocussing)

;spnam4: Bip720,100,10.1

;sp7: f2 channel - shaped pulse 180 degree for refocussing

;spnam7: Crp60comp.4 (Crp80comp.4)

;p1 : f1 channel - 90 degree high power pulse

;p2 : f1 channel - 180 degree high power pulse

;p3 : f2 channel - 90 degree high power pulse

;p14: f2 channel - 180 degree shaped pulse for inversion

;p16: homospoil/gradient pulse

;p21: f3 channel - 90 degree high power pulse

;p22: f3 channel - 180 degree high power pulse

;p24: f2 channel - 180 degree shaped pulse for refocussing

;p28: f1 channel - trim pulse

;p29: gradient pulse 3 [300 usec]

;p39: f2 channel - 180 degree shaped pulse for refocussing

; Bip720,100,10.1 (160us at 600.13 MHz)

;d0 : incremented delay (2D) [3 usec]

;d1 : relaxation delay; 1-5 * T1

;d2 : 1/(2J)XH

;d4 : 1/(4J)XH

;d11: delay for disk I/O [30 msec]

;d16: delay for homospoil/gradient recovery

;d62: length of block between decoupling pulses : = aq/l0 [< 20-25 msec]

;d63: = d62/2

;cnst2: = J(XH)

;l0 : number of blocks during acquisition time

; adjust to get d62 as required

;inf1: 1/SW(X) = 2 * DW(X)

;in0: 1/(2 * SW(X)) = DW(X)

;nd0: 2

;ns: 1 * n

;ds: >= 16

;td1: number of experiments

;FnMODE: echo-antiecho

;cpd2: decoupling according to sequence defined by cpdprg2

;pcpd2: f2 channel - 90 degree pulse for decoupling sequence

;for z-only gradients:

;gpz1: 80%

;gpz2: 20.1% for C-13

;gpz3: 25%

;gpz5: 25%

;use gradient files:

;gpnam1: SMSQ10.100

;gpnam2: SMSQ10.100

;gpnam3: SMSQ10.50

;gpnam4: SMSQ10.50

;preprocessor-flags-start

;LABEL_CN: for C-13 and N-15 labeled samples start experiment with

; option -DLABEL_CN (eda: ZGOPTNS)

;preprocessor-flags-end

;$Id: $
